# Factors Influencing Parental Engagement in an Early Childhood Obesity Prevention Program Implemented at Scale: The *Infant Program*

**DOI:** 10.3390/nu10040509

**Published:** 2018-04-19

**Authors:** Penelope Love, Rachel Laws, Eloise Litterbach, Karen J. Campbell

**Affiliations:** 1 Institute for Physical Activity and Nutrition, School of Exercise and Nutrition Sciences, Deakin University, Geelong 3222, Australia; r.laws@deakin.edu.au (R.L.); karen.campbell@deakin.edu.au (K.J.C.); 2 Centre of Research Excellence, Early Prevention of Obesity in Childhood (EPOCH), Deakin University, Geelong 3222, Australia; 3 School of Exercise and Nutrition Sciences, Deakin University, Geelong 3222, Australia; e.litterbach@deakin.edu.au

**Keywords:** childhood obesity, parental engagement, maternal and child health, research translation, implementation, infant feeding, active play

## Abstract

The ‘early years’ is a crucial period for the prevention of childhood obesity. Health services are well placed to deliver preventive programs to families, however, they usually rely on voluntary attendance, which is challenging given low parental engagement. This study explored factors influencing engagement in the *Infant Program*: a group-based obesity prevention program facilitated by maternal and child health nurses within first-time parent groups. Six 1.5 h sessions were delivered at three-month intervals when the infants were 3–18 months. A multi-site qualitative exploratory approach was used, and program service providers and parents were interviewed. Numerous interrelated factors were identified, linked to two themes: the transition to parenthood, and program processes. Personal factors enabling engagement included parents’ heightened need for knowledge, affirmation and social connections. Adjusting to the baby’s routine and increased parental self-efficacy were associated with diminished engagement. Organisational factors that challenged embedding program delivery into routine practice included aspects of program promotion, referral and scheduling and workforce resources. Program factors encompassed program content, format, resources and facilitators, with the program being described as meeting parental expectations, although some messages were perceived as difficult to implement. The study findings provide insight into potential strategies to address modifiable barriers to parental engagement in early-year interventions.

## 1. Introduction

The ‘early years’, more recently defined as the first 1000 days (conception to 24 months), is widely acknowledged as a crucial period in laying the foundation for life-long learning and development [1,2], and more specifically, for the prevention of childhood obesity [3]. Globally, an estimated 41 million children aged under five are overweight or obese [4]. In Australia, 20% of children aged 2–4 years are overweight or obese [5], with predictions that this could reach 33% by 2025 [6]. One in three children living in lower socioeconomic areas (33%) are overweight or obese compared with those living in higher socioeconomic areas (19%), while levels are comparable across urban (26%) or regional (27%) areas [7]. Overweight, obesity and related comorbidities track into adulthood. The health consequences of these conditions in childhood are thus well expressed across the life course, such as diabetes, heart disease, some cancers, respiratory disease, mental health and reproductive disorders [8]. 

Parents and carers play a significant role in influencing the health and development of their children, and supporting parents to develop their knowledge, skills and confidence regarding child health and development is considered an important public health strategy [9,10,11]. Family-based health services are well placed to deliver preventive interventions in the form of group-based programs and alternative modalities [12,13,14,15]. Unlike treatment services, however, preventive interventions usually rely on voluntary attendance. Low engagement is common, resulting in program benefits being diluted and program delivery being costly to the organisation [16,17,18,19,20,21]. 

Australian parents are highly receptive to information and support during the first year of their infant’s life, making an average of 11 visits to their general practitioner and 14 visits to their maternal and child health nurse, with the majority of these unrelated to illness [22]. The universal free maternal and child health service available in Victoria, Australia, provides 10 “Key Age and Stage” consultations between birth and age 3.5 years [23]. This service is highly accessed, with >90% attendance rates for the 2-, 4- and 8-week and 4-month appointments; 80% attendance rates for the 8- and 12-month appointments; and >60% attendance rates for the 2- and 3.5-year appointments [24]. Parental engagement is a complex phenomenon, broadly defined as “getting participants to come” [25]. A range of terms are used to describe the “engagement trajectory”, including recruitment and enrolment; participation and involvement; attendance and retention; and completion, termination and attrition [17,23,26,27,28,29]. Wen (2016) also describes engagement as comprising two broad dimensions of quantity (attendance rates or dosage) and quality (participant interactions with the program), suggesting that the quality of the interactions may be a stronger predictor of program outcomes than the quantity of participation [26]. Studies investigating parental engagement in family-oriented prevention programs and community-based home visiting programs have begun to examine patterns and variations of attendance, to determine impacts in relation to specific time points of engagement [25,26]. This has led to the differentiation of participants into categories of attendance, such as “early terminators” (completing 25% or less of the program) [27], “completers” (completing 80% or more of the program) and “non-completers” [30], or “non-initiators”, “initiators”, “late dropouts”, and “low and high sporadic attenders” [17]. 

Examining patterns of attendance is considered important to determine if there are sensitive periods when attrition is more likely to occur [27], and the reasons for this [26,29]. Understanding the different barriers and enablers that influence parental engagement, especially for those most in need of services but least likely to access them [21], is valuable. Such findings can inform the development of appropriate strategies to enhance program reach and sustained delivery, thereby improving the cost-effectiveness and health impacts of the intervention or program [16,26,28].

Enablers and barriers to engagement are most commonly categorised into personal, organisational and program factors. Personal (family, parent) factors include socio-demographics; physical, financial and emotional resources; and social supports [18,19,20,21,27,31,32,33,34,35,36,37]. Organisational (service provider) factors include workforce capacity; organisational culture and leadership; and implementation readiness [18,32,36]. Program (process, therapist) factors include the content, delivery and design of the intervention [19,20,27,32,33]. Kelleher (2017) sub-categorises barriers further into modifiable and non-modifiable factors, with modifiable factors regarded as potentially preventable [16]. 

Studies of the enablers and barriers to engagement have most frequently been conducted in the context of children with behavioural [13,25,30,32], mental health [20], or weight management issues [10,16,17,33,38], or children who have complex medical concerns [9] or vulnerable families [29]. Studies investigating first-time parents have focused on neonatal issues such as prematurity and neo-natal follow-up [18], home visitation programs [26,27], randomised controlled trials [39,40], and the perspectives of researchers [41]. Studies exploring the engagement of first-time parents with preventive health programs focusing on establishing healthy lifestyle behaviour in early infancy appear to be limited.

This study is an exploration of the factors influencing the engagement of first time parents in an early childhood group-based obesity prevention program, from the perspectives of both program service providers and parents across all levels (non, low and high) of program attendance. For the purposes of this study, parental engagement is defined as program recruitment (promotion and enrolment) and program retention (ongoing attendance). 

## 2. Materials and Methods

This study used a multi-site qualitative exploratory approach, underpinned by a contextualist epistemology, where knowledge emerges from and is situated within the contexts of the data [42]. Understanding and interpreting the data can therefore be influenced by the roles and backgrounds of the researchers and should be made explicit. P.L. and E.L. conducted all interviews. P.L. undertook full data coding and thematic analysis, with cross-checking for coding consistency and input into final theming by R.L. and K.C. All researchers are mothers and qualified dietitians/nutritionists working within a research context. E.L. is a research assistant with experience in researching infant and child nutrition practices. P.L. is a postdoctoral researcher with experience in the implementation of public health nutrition interventions at a community level, and with no involvement in the development of the *Infant Program*. R.L. is a postdoctoral researcher with experience in research translation, with specific involvement in evaluating the community-wide implementation of the *Infant Program*. K.C. is a professor and chief investigator of the *Infant Program*, responsible for its development, randomised control trial, community-wide implementation, and ongoing evaluation. Ethical approval for this study was obtained through Deakin University (HEAG-H 160_2016) and the Victorian Department of Education and Training (DET 2016_003167). 

### 2.1. Study Context: The Infant Program

The *Infant Program* is a low dose, efficacious, group-based program for first time parents [43]. Six 1.5 h sessions are delivered at three-monthly intervals, commencing when infants are around three months of age. The program is underpinned by parenting support theory and utilises an anticipatory guidance framework to guide content delivery focused on feeding and active play [44]. The development of the program was informed by two systematic reviews [45,46] and a cluster randomised controlled trial (RCT) [43]. Findings from the RCT found the program improved maternal nutrition knowledge and preferred feeding behaviours [47]; maternal dietary patterns [48]; child’s dietary and sedentary behaviours [43]; child diet quality [49]; and water and vegetable intake in the sub-groups [50].

The *Infant Program* has been implemented at a community level in a number of Local Government Areas (LGAs) in Victoria, Australia since 2014, supported by ongoing research regarding the translation and extension of the program into routine service delivery [51,52]. The main source of program referral is the maternal and child health nurse (MCHN) via the universal, free “Key Ages and Stages” appointments provided to all mothers across Victoria. Recruitment to the program is typically through first-time (new) parent groups, with LGAs using either an opt-in or opt-out approach. The opt-in approach involves enrolling interested parents for the first session and parents self-enrolling for subsequent sessions. The opt-out approach involves enrolling interested parents for all sessions at the start and parents indicating their attendance in response to text notifications. All LGAs send reminder text notifications to registered parents ahead of each scheduled session. 

### 2.2. Study Site Selection

Three LGAs were purposefully selected from seven LGAs running the *Infant Program* at the time of the study. All agreed to participate, and were representative of diverse geographic locations (urban, regional and rural). All LGAs had implemented the program since 2015, with community sites adapting aspects of program implementation to suit local organisational needs (Table 1).

### 2.3. Recruitment of Program Service Providers

Organisational and participant consent was obtained prior to conducting 30 min telephone interviews with program service providers at each study site. No incentives were provided. 

### 2.4. Recruitment of Program Participants

All parents who had registered interest in attending the *Infant Program* between 2015 and 2016 were invited via text notification to participate in the study. Text message content was provided by the researchers, and disseminated by program service providers at each study site. Two rounds of text messaging were scheduled across all sites (one month apart), however only one round occurred for the regional site as their program funding and implementation ceased during the study period. Interested parents were directed to a short online survey to collect data on program attendance, level of education, home language, postal code, and consent to participate in a 30 min telephone interview. To examine engagement in relation to patterns of attendance, program attendance was categorised into three levels; ‘non-attendance’, defined as having registered interest but not attended any program sessions; ‘low attendance’, defined as attending three or fewer of the six program sessions; and ‘high attendance’, defined as attending four or more of the six program sessions. Parents consenting to an interview were stratified by program attendance then contacted by the researchers to arrange a convenient date and time. All parents received a $20 grocery gift voucher on completion of an interview.

### 2.5. Qualitative Interviews and Thematic Analysis

All interviews were conducted telephonically by two researchers (P.L. and E.L.) using semi-structured interview guides. Program service providers were asked about their role within the organisation and in program implementation, about perceived barriers and enablers to program promotion, enrolment, delivery and retention of participants. Parents were asked how they had heard about the *Infant Program*, what prompted them to enrol, their sources of information and support regarding infant feeding, and their expectations of the program. Non-attendees were asked about reasons for not attending and how these barriers may have been addressed. Low and high attendees were asked about their expectations of the program, reasons for intermittent attendance and how these barriers may have been addressed. High attendees were asked about reasons for continued attendance.

Audio-recorded interviews were professionally transcribed verbatim. A sample of interviews were initially coded by three researchers (P.L., R.C. and K.C.) to check for coding consistency, then all interviews were coded by P.L. using NVivo. Thematic analysis was undertaken, with initial codes based on phrases emerging systematically and repetitively across the data, and consensus on final themes developed in agreement between two researchers (P.L. and R.L.). A case comparison analysis approach was used to identify similarities and differences between barriers and enablers for non, low and high program attendees, and the perspectives of program service providers across the three study sites. 

## 3. Results

### 3.1. Description of Study Participants

#### 3.1.1. Program Service Providers

All program service providers (*n* = 10) across the three study sites were invited to participate, with nine being interviewed; three identified as program coordinators, responsible for operational and logistical program implementation; and six identified as program facilitators, responsible for program content delivery. One program facilitator was male, with all other program service providers being female. Program coordinators were employed in team leader (*n* = 1), health promotion manager (*n* = 1) and maternal and child health nurse (*n* = 1) roles, and program facilitators included a social worker (*n* = 1), dietitians (*n* = 2) and maternal and child health nurses (*n* = 3).

#### 3.1.2. Program Participants

Text notifications were sent to 500 parents who had previously participated in the program between 2015 and 2016. Fifty-three parents responded to the online survey (RR10.6%), with a lower response rate for the regional site (urban *n* = 230; RR11.3%; regional *n* = 90; RR7.8%; rural *n* = 180; RR11.1%). All respondents were contacted for an interview; three declined, 18 were not contactable, and 32 (60.4%) were interviewed, representing all study sites, all categories of program attendance and all levels of education. The majority of interviewees were English-speaking (96.9%) with university (40.6%) or apprenticeship (34.4%) qualifications. Equal numbers of interviewees had participated in the program as either high (*n* = 15,) or low (*n* = 15, 46.9%) attendees, with few interviewees (*n* = 2, 6.3%) being non-attendees. A comparison of participant characteristics indicated that those selected for interview were representative of the total sample expressing interest to participate in the study (Table 2). 

### 3.2. Factors Influencing Parental Engagement

**Figure 1:**
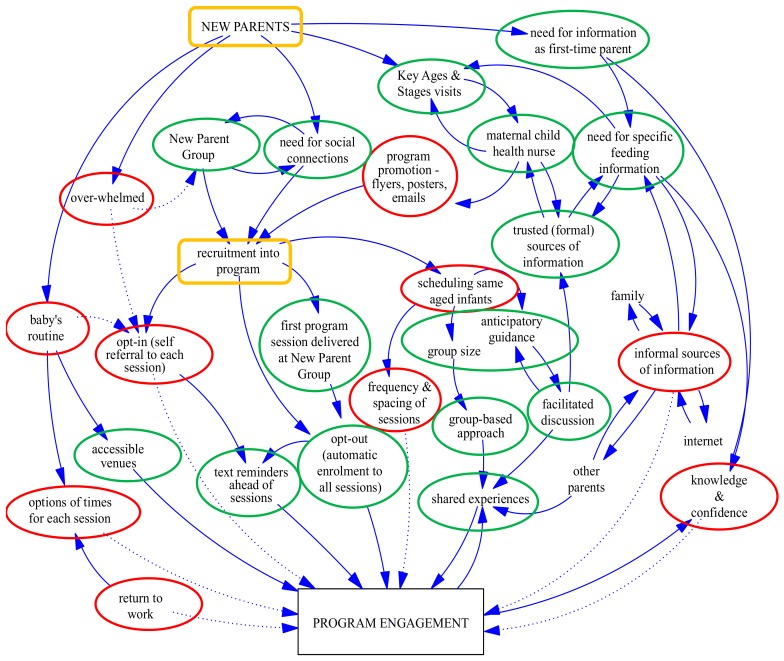
Factors influencing parental engagement: perspectives of program service providers and program participants. (Dotted arrows indicate factors leading to non/low program attendance; yellow blocks indicate themes; red circles indicate barriers; green circles indicate enablers).

Figure 1 reflects the complexity and interrelationship of the factors influencing engagement as identified by parents and program service providers. Two emerging themes were apparent—the transition to parenthood and program processes—which are outlined briefly here and described in more detail below. As new parents, the *transition to parenthood* heightened the need for information, especially on infant feeding, and the need to share experiences with other parents. new parent groups and maternal and child health services fulfilled these needs as trusted sources of information and opportunities to form social connections; and were therefore regarded as good opportunities to recruit parents into the program. Accessing information through other (informal) sources, such as family and social media, and adjusting to the baby’s routine, were likely to reduce parent engagement. *Aligning program processes* to meet the needs of parenthood was important to enhance engagement, such as offering program sessions on different days and at different times to accommodate the baby’s routine and parents returning to work. Engagement was also enhanced when the program content and delivery style created an opportunity for group discussions and strengthened social connections (Figure 1). 

A detailed overview of these themes, and the enablers and barriers to engagement within each theme, is provided below and in Table 3. Where relevant, interviewee quotes are provided to illustrate specific perspectives. Table S1 contains a comprehensive set of interviewee quotes. 

#### 3.2.1. Theme: Transition to Parenthood

The transition to parenthood was described as enabling program attendance due to a new parent’s need for information, specifically information regarding infant feeding, and a need to form social connections with other parents. A primary enabler of attendance, common to non, low and high attendees, was “*the need for information as a first time mother*”. Low and high attendees expressed an additional need for specific information and reassurances about *infant feeding* (breastfeeding, food textures, types of foods, food allergies, recipes, meal plans). Other topics of interest were sleeping and active play, with high attendees also interested in information about home safety, choking and First Aid; and low attendees enquiring about teething, infectious diseases, and preparing their infant for the arrival of a sibling. High attendees also described the need to “*socialise with other parents*” and “*to share experiences*”, with the program providing this opportunity. Attending the program was described as positively benefitting the transition to parenthood.

*“It’s something I would recommend [to new parents]. There were a lot of things that I was, like, ‘oh now we can do this’ and ‘I should be doing this’. It was really good. I took a lot of notes”*

*(low attendee, urban 12)*

*“I learned a lot from it. I knew nothing about solids or how to introduce them or when to introduce them. I walked away with a clearer idea … That was the first place that I really learned about that. If I hadn’t gone there I don’t know where I actually would have learned that except from a relative” *

*(high attendee, urban 9)*

*“It was just nice to have somebody there who was going through the same thing really … you didn’t feel judged because you were all going through that, yeah, having that level of friendship really helped” *

*(high attendee, rural 14)*

Parenthood posed some barriers to program attendance in the form of low levels of parental self-efficacy, receiving conflicting information and guidance from informal sources (such as family and the internet), and returning to work. Non-attendees described parenthood as an “*overwhelming*”, “*daunting*” and “*stressful*” period, hampering attempts to self-refer or attend program sessions. The desire not to interrupt baby’s routine (especially sleeping) was a commonly described barrier across all categories of attendance, further reducing the likelihood of self-referral and attendance. Non- and low attendees, and parents with lower levels of education, described *a reliance on informal sources of information*, such as family members, friends with children, the internet, and social media, which was viewed as a substitute for program attendance.

*“I know it’s really good to get out of the house, but I just felt—and there’s so much pressure to do it. I actually felt like a bit of a failure, because I wasn’t getting there” *

*(non-attendee, regional 2)*

*“I’d attend if it wasn’t his sleep time. I wasn’t going to wake him to go” *

*(low attendee, regional 3)*

Across all study sites, program service providers identified two time periods when program attendance declined; session 3 (infants about nine months) and session 4 (infants about 12 months). Program service providers and low attendees concurred that the main reason for program attrition around nine months was a *growing sense of confidence as a parent*, especially once infants had transitioned to solid foods; and with *mothers returning to work* at around 12 months. For high attendees, returning to work was also described as resulting in a loss of social connections with other mothers, which reduced their likelihood of attending the remaining (15- and 18-month) program sessions. 

#### 3.2.2. Theme: Program Processes

A number of enablers and barriers to program attendance were described in relation to aspects of program promotion, referral, recruitment, enrolment, scheduling, and delivery.

- Program promotion and referral

Given the high uptake of the ***‘****Key Ages and Stages’ appointments* by new parents, program service providers considered the maternal and child health services to be an appropriate mechanism for program promotion and referral. Non, low and high program attendees concurred with this, describing the maternal and child health service as a “*highly trusted source of information and referral*”*. Limited program awareness* was considered a barrier by program service providers and participants across all categories of attendance. Parents suggested that promotion of the program should ideally be before the birth of the baby to create early awareness and encourage attendance ahead of (feeding) issues arising, and that this could be done via usual appointments with midwives, general practitioners, obstetrics and gynaecological services during pregnancy. Program promotion through local government websites, libraries, lactation consultants, child care centres, social media, community events and local newspapers was suggested, with non- and low attendees recommending that promotional material should include information about the program content.

- Program recruitment

Across all study sites, program service providers considered recruitment through existing *new parent groups* as highly efficient, providing a captive audience with existing social connections. Low and high attendees also described new parent groups as important in establishing trusted friendships, which encouraged program enrolment and attendance.

*“I think it’s probably the best way, I would say between 80 and 90% of parents going to those groups would actually express interest … and that cut out a lot of the admin of me trying to follow-up with people, because they were already coming together”*

*(program service provider, regional 5)*

*“It probably helped hearing about it in the [new parent] group, because I think I went along to at least the first session with some other mums from the same group”*

*(low attendee, urban 13)*

The recruitment of new parents who were not members of new parent groups was a challenge for program service providers, and a concern shared by some parents, with the suggestion that multiple promotional channels be used. Some parents commented on the program only being offered to first-time mothers, suggesting that multiparous women be able to attend as “*every child is different*”.

*“I’ve got a 2 year gap between my two, so my little one is 6 months old, and there are things that you forget … Perhaps a little refresher course” *

*(low attendee, rural 18)*

*“It would be great to be able to attend as a second or third time mum for a refresher”*

*(high attendee, rural 23)*

Across all study sites, program service providers were concerned about reaching vulnerable parents, specifically Aboriginal parents, culturally and linguistically diverse (CALD) parents, adolescent mothers and disadvantaged parents, who were unlikely to attend new parent groups. Working in partnership with other organisations already accessing these communities was a common suggestion to extend program reach, although this was also considered to be time intensive, requiring additional financial and human resources to adapt program content and delivery.

*“I don’t think we’re doing a fabulous job of reaching, perhaps, vulnerable families as well as we could be, because they’re not necessarily the families that are going to participate in a new parents group for whatever reason … We’ve probably got the groups there that we could partner with, and I think that’s been one of my really big learnings—don’t try and do it yourself, partner with the agencies that are already in contact with those groups, because there’s absolutely no point in setting it up and doing it yourself” *

*(program service provider, rural 8)*

*“Often what it takes to engage those more vulnerable families is a lot more work. A lot more time—probably more funding in terms of you know—you’d often need an interpreter. Sometimes you would need an extra staff member, potentially to run a program or to run a session if you had families there who were, you know, English as a second language” *

*(program service provider, regional 6)*

- Program enrolment

An *opt-out enrolment* approach, with participants automatically enrolled in all sessions from the start, was described positively by program service providers and high attendees. In addition, the integration of the first session of the *Infant Program* as the last session of new parent groups provided immediate exposure to the program content and format, encouraging program enrolment. While an *opt-in (self-referral) enrolment* approach was considered by some program service providers as providing flexibility and a degree of accountability to parents, most described this as an administratively burdensome task. Low attendees also described self-referral as an activity likely to be forgotten given the spacing of the program sessions at three-monthly intervals. *Reminder notifications*, sent by text message at least a week ahead of a scheduled session, were considered useful, especially given the spacing between sessions and changes to the baby’s routine. Parents suggested that the content of the forthcoming session be promoted with the reminder notification to enhance interest and attendance.

- Program scheduling and delivery

For all study sites and across all attendance categories, *program venues* were considered safe, comfortable and accessible (by car, public transport and on foot), with adequate parking and space for prams. Providing *multiple session options* on different days/times was a challenge for program service providers, constrained by the availability of staff and the need to ensure an adequate group size with infants of the same/similar age. Parents reported that limited session options reduced flexibility to attend when circumstances such as the baby’s routine changed, or to accommodate working parents, including fathers.

*“I’m still sort of thinking how we could do that … engage a bit better with fathers … as far as what their working arrangements are” *

*(program service provider, regional 5)*

*“It’s difficult because your baby obviously does sometimes change their sleep patterns and that sort of thing … It would have been nice to have a bit more options for time” *

*(low attendee, urban 1)*

*“I thought maybe it would be nice to have two sessions, one in the afternoon and one in the morning, not necessarily on the same day, because that might be really difficult, but maybe in the same week. Just to give parents an option … even after-hours so dads might be able to come”*

*(high attendee, urban 5)*

The scheduling of participants to ensure an *appropriate group size with infants of the same/similar ages* was described as an administrative challenge across all sites, and particularly problematic for (rural) areas with low birth rates. Reaching vulnerable parents through existing infrastructure, such as supported playgroups, added further complexity to scheduling, as these groups comprised multiparous mothers and infants of varying ages. Scheduling infants of approximately the same age within groups, however, was appreciated by low and high attendees as it enabled the delivery of program content using an *anticipatory guidance approach*, providing age-appropriate, timely information.

*“I think getting a critical mass of babies at the right age is actually quite difficult … and that is the case for a number of [rural] areas. I think you need a really good system for that, otherwise it becomes all-consuming” *

*(program service provider, rural 8)*

*“It was going to be age appropriate … If someone had have just thrown a book at us when the kids were born, it wouldn’t have been helpful”*

*(high attendee, rural 11)*

The *group-based format* of the program was regarded as an enabler by program service providers and low and high attendees. This format was viewed as providing a relaxed, friendly environment that encouraged peer discussion, social connections and trust, with suggestions that a group size of 10–15 parents was appropriate for good group dynamics.

*“The real advantage of these groups is that you do build that trust, and families do feel supported”*

*(program service provider, regional 5)*

*“I’m a bit of a shy person, so I liked that I could just sit there quietly but still feel part of it. There was no pressure. People asked questions; I didn’t have to, because other people asked valid questions. Then there were conversations going on. It was nice to hear that everyone is on the same page as you”*

*(low attendee, urban 11)*

*“The main thing I got out of it was listening to other people, their experiences … some real reassurance I suppose and knowing that you were in the same situation as other people, having the same week as other new mums”*

*(high attendee, rural 11)*

Across all categories of attendance, parents with higher levels of education showed a preference for *formal sources of information*, with group facilitators described as “*trusted*”, “*knowledgeable*” and “*approachable*”. Dietitians were valued for their specific nutritional knowledge and maternal and child health nurses for their expertise in child health. While parents appreciated the technical knowledge and professional *expertise of group facilitators*, they expressed a need for them to have experience as a parent, or at least some practical experience working with infants.

Parents enjoyed external speakers (such as dentists or first aid instructors), and practical “*hands on*” advice, especially recipe ideas. The program resources were valued by parents, with a preference for hardcopy handouts at sessions “*to refer to at home*” and digital sources that could be accessed if unable to attend.

*“It would have been good to have extra information emailed to you if you couldn’t attend a session”*

*(high attendee, regional 4)*

*“Perhaps it could be recorded … like if someone misses a session or you want to re-look at something … or be available online”*

*(high attendee, rural 8)*

*“I’ve stuck it [handout] on the fridge … from the first session and I still refer to it. It’s good to have something to take away because it’s just really overwhelming”*

*(low attendee, urban 10)*

Certain aspects of the *program content* appeared to be a barrier for low attendees with higher levels of education, who considered certain messages to be either “*too prescriptive*”, “*too general*” or a duplication of the information received at their maternal and child health appointments. Program content regarding screen time recommendations was recalled most often as behaviour that would be difficult to implement given the insidious use of digital technology.

*“They [participants] were often quite surprised to hear the recommendations were no screen time up to two years, and there were lots of questions about ‘well what do we do as parents then for our own sanity?’”*

*(program service provider, regional 6)*

*“I guess I was a bit disappointed, it didn’t really tell me anything that I didn’t already know. I guess I expected to get more hands on experience out of it. I felt like it was really just general healthy eating guidelines” *

*(low attendee, regional 3, university)*

*“Parts of it were great and I got a lot out of it but there were parts where I just thought, ‘I feel a bit judged now’. It was like ‘this is the recommendation’”*

*(low attendee, urban 12, university)*

Linked with periods of program attrition, especially after session 4, suggestions were made by program service providers and program regarding the *frequency and spacing of sessions*, with a preference for fewer sessions (2–4 rather than 6), spaced closer together (“*perhaps monthly*” rather than three months apart), completed earlier (within 12 as opposed to 18 months), and linked to reliable sources of digital information.

*“Three months is a long time in between … if we were to condense it into a shorter timeframe that would be helpful because really, you know, they really need the advice maybe monthly”*

*(program service provider, regional 5)*

*“We weren’t getting many 15 and 18 month attendees … now we go to the 12 month and we send them out a text message with a link to the [program] website so they can still have access to the information, and they’re more than welcome to call up” *

*(program service provider, rural 7)*

## 4. Discussion

This is one of few studies examining the parental engagement specifically of first-time parents in an early childhood obesity prevention program from the perspectives of program service providers and program participants with varying patterns of attendance. The factors affecting parental engagement in the *Infant Program* were related to two interconnected themes: the *transition to parenthood* and *program processes*; and across these themes, enablers and barriers were linked ***to*** personal, organisational and program factors. 

Personal factors included *parental knowledge and self-efficacy*; *social connections*, and *sources of information and support*. A heightened need for knowledge and assurances, especially infant feeding information, and opportunities to share experiences and develop social connections, were described as primary motivators for enrolling in the *Infant Program*. These enablers, however, were thwarted if parenthood was experienced as stressful and overwhelming, adjusting to the baby’s routine was challenging, and informal sources of information were substituted for program attendance. 

Program attendance appeared to diminish with the acquisition of knowledge and growing confidence as a parent, especially once infants had transitioned to solid foods; and early program termination appeared to be linked to a loss of social connections with the group once parents returned to work. The link between reduced program attendance and increased parental self-efficacy is consistent with other studies where parents with a lack of confidence in their parenting abilities and a belief in the program benefits were more likely to see the relevance and importance of program attendance [12,16,20,32,33,35]. The evidence also supports the benefits that social connections provide in enhancing program attendance, describing them as perhaps “more important than the information parents receive in the group” [12], as it creates a sense of solidarity [53], reduces feelings of social isolation and fosters confidence [11]. 

Increased parental self-efficacy as a result of attending the *Infant Program* is considered a success, despite it potentially leading to reduced program attendance. To this end, the program encourages the attendance of sessions by others involved in the care of the infant (such as fathers, partners, grandparents) to build a shared understanding of program messages and increase the likelihood of their implementation. Research also continues regarding the efficacy of social media (web-based resources, apps and Facebook) as a means of directing *Infant Program* participants to reliable sources of information [52,54,55].

A reliance on informal sources of information and support from family, friends with children, the internet and social media, appeared to become a substitute for program attendance, especially for program participants with lower levels of education. Participants with higher levels of education appeared to seek out ‘reliable’ information and showed a preference for formal sources of support, in particular the maternal and child health nurse. These findings are supported by other studies, where the preference to access formal sources of information was found to increase when parents developed a good relationship with the source [16,20,21,34,56]. It is suggested that while the use of technology (internet websites, forums and apps) to access health information by parents is ubiquitous, mothers with higher levels of education are still likely to use ‘authoritative online sources’, and mothers with lower levels of education are more likely to use discussion forums and social media [57,58,59]. Being comfortable using technology is an important factor in determining the use of online resources by parents [59], and given the multitasking nature of parenthood, online resources must be easily accessible and readily understood [58]. 

Organisational factors affecting parental engagement included *program promotion and referral; recruitment*; *enrolment, scheduling and access*. Promotion and referral through the maternal and child health service via routine ‘Key Ages and Stages’ consultations was regarded as an efficient mechanism, given the high uptake of this service and frequency of contact. Broader program promotion, especially prior to the birth of the baby, was recommended, however, to increase program awareness. A lack of program awareness, as identified in this study, is frequently cited as a practical barrier to engagement [21,32,36], often related to insufficient or ineffective advertising and resulting in reaching only those who proactively seek support [19]. Effective promotion is described in the literature as multi-channel (leaflets, posters, internet, local newspaper/radio stations, posted newsletters, and social media parenting forums), with clear content information that conveys the tangible benefits of the program, using text and images relevant to the target audience [19]. Studies also support the suggestion of program promotion prior to the birth (through ante-natal care) and early after birth (through home visits/support services/parenting groups) [32,55,60], to provide the opportunity to address potential personal barriers to engagement [15,18,20]. 

Recruitment to the *Infant Program* via new parent groups was considered an enabler, providing access to parents with infants of the same/similar ages and with existing social bonds. The source of program referral is described in the literature as an important contributor to the likelihood of attendance, with referrals from trusted sources [19] (such as a maternal and child health nurse) positively influencing participant impressions of the relevance of a program [32,33]. Direct and personalised recruitment, via word of mouth, emails, phone calls and text message is therefore regarded as effective when delivered by people who have an established relationship with the parent [19]. Informal social networks are therefore encouraged as a means of extending recruitment options and positively influencing social discourse about the program topics [32].

Organisations were concerned, however, about how best to recruit parents who did not attend new parent groups, especially vulnerable parents, stating that organisational partnerships and adequate workforce resources were necessary to achieve this. These concerns are mirrored in other studies, with recommendations that related services work together to create strong interagency collaborations that provide multiple referral routes [19], targeted recruitment [32] and cross-promotion, co-design and shared delivery of the program [37]. These are important considerations given the high rates of obesity among families of low socio-economic position.

The link between program attrition and the way in which participants are enrolled into programs is also of interest, especially in the context of voluntary program participation, which relies heavily on intrinsic motivation [27]. Study sites using an opt-out approach, where participants were enrolled in all sessions at the start, appeared to have higher patterns of attendance compared to those using an opt-in approach, which relied on the participant to self-enrol for each session. Opt-out enrolment processes have been found to be beneficial in combination with highlighting program benefits (such as enhanced knowledge, skills and social connections) [16], prompting attendance with timely reminder notifications, and ensuring that program activities are attractive to parents [37].

Scheduling groups with adequate numbers of parents with infants of the same/similar age was an ongoing challenge, especially for areas with low first-time parent birth rates. This limited the ability to offer a choice of session days and times, and in some cases resulted in program sessions accommodating a large age range (such as 9- and 12-month old babies). Providing a narrow range of program times has been reported to limit the attendance of parents with busy schedules [32], time constraints [19], and those returning to work [21,32]. Attendance of only one parent can also place barriers on the use of program information if this information is likely to conflict with or contradict the views of the non-attending parent [32]. This is of particular relevance given that all program participants in this study were mothers, and only one program service provider was male. The literature describes fathers as being poorly engaged by mainstream services due to the framing of parenting programs around mothers [36], program delivery usually occurring in a female-oriented environment [21], and usually during working hours [37]. Evidence supporting the influence of fathers on the health behaviour of their young children is increasing, however, with fathers regarding themselves as playing an important role in promoting and supporting healthy eating and physical activity, and expressing a desire to have access to reliable information [61]. Developing and delivering programs to accommodate parenting couples, extended family members and other carers is an important future consideration, as is the delivery of program sessions by male and female facilitators.

The program venues were described by study participants as accessible, safe and comfortable, thus enabling program attendance; with high patterns of attendance within the rural study site (62.5%). Physical access to programs is described as an important enabler in many studies, especially in rural areas where public transport may be limited or costly [19,32,33,36]. Apart from conveniently located venues that are accessible by public transport, practical considerations, such as ample parking and space for prams [19,21], and perceptions of the safety, confidentiality and quality of the venue [36] have also been found to influence the social experience and engagement of participants. 

Program factors affecting engagement encompassed the program *content, format and resources*; and *delivery by trusted individuals*. Program content focused predominantly on infant feeding and active play and therefore met the expectations of participants, however, messages perceived by participants as difficult to implement, such as screen time recommendations, were described as *‘prescriptive’* and *‘judgemental’*, especially by program participants with low patterns of attendance. Similar findings have been seen among low program attendees expressing fears of being judged by others or not possessing the necessary skills [19,36] and service providers potentially evading these ‘difficult’ topics for fear of disengaging parents [62]. The evidence suggests that group facilitators use effective communication and a supportive, non-judgemental, interactive style to involve participants and to overcome this challenge [21,32]. Similarly within the *Infant Program*, participants enjoyed the inclusion of external speakers (such as dentists or first aid instructors) and peer advice (recipe ideas) to augment program information. The group-based approach was described positively by program participants and program service providers, encouraging group discussions and providing opportunities to form social contacts. Nobles and co-authors describe group size as a strong predictor of parental engagement, and found that groups of fewer than 20 participants, as opposed to one-on-one, encouraged social interaction and promoted a sense of belonging [17]. 

The important role played by group facilitators in creating an environment for social bonding is closely linked to the personal need of participants to share experiences and seek reassurance, often reducing feelings of isolation and normalising concerns [16]. The ability to form supportive relationships with participants [27,36], to ensure that participants feel included in the group, and to encourage the mutual sharing and positive reinforcement that builds confidence and parental self-efficacy, are viewed as important group facilitator qualities to prevent program attrition [19,32], in addition to the necessary training and expertise [21,33] regarding program content. Apart from professional skills, this study found a preference by program participants for group facilitators with personal experience in caring for infants; a finding supported by Koerting and co-authors [19]. 

Across the study sites, the provision of experienced professionals to facilitate groups was a challenge when workforce capacity was stretched by existing clinical workloads. The integration of program implementation as routine practice, thereby attracting organisational resourcing, was described by one study site as a means of addressing workforce capacity. Studies note that the turnover of staff is likely to affect relationship-building and impact program attendance, suggesting the continuity of group facilitators throughout program delivery [16,27]. 

These findings align with those identified by others, where parental engagement was enhanced when the program met the expectations and needs of the participants [19], the program content had clear objectives and was underpinned by a strong theoretical base, and program delivery was flexible, in order to accommodate busy and hard-to-reach parents [32].

## 5. Study Implications

The study findings provide insights into the factors influencing parental engagement in an early childhood obesity prevention program, and therefore also highlight the changes needed to address modifiable barriers to engagement. While numerous studies focus on the influence of personal factors on parental engagement, there is growing acknowledgement that the majority of modifiable factors are likely to be found within the program and/or its implementation [16,27,37]. The strategies suggested below therefore focus on enhancements to address program and organisational barriers, which, if implemented, would help to address cumulative personal barriers (Box 1).

Future research considerations include obtaining perspectives from a larger sample of non-attendees, as well as fathers and parents of different ethnicities. Understanding the predictors of engagement for these cohorts could provide additional insights into the strategies needed for equitable program access and uptake, thereby extending the reach, cost-effectiveness and health benefits of the program.

Box 1 **Suggested strategies to enhance parental engagement in the *Infant Program*.**

1. Broader program promotion through multiple channels, with more information about program content and benefits
2. Earlier program promotion during pregnancy and through initial home visits/home support service, with an opportunity to address potential barriers to attendance
3. Continued program promotion and referral through trusted sources of information and support, especially maternal and child health services with routine contact
4. Cross-promotion and referral through interagency collaborations, especially to reach vulnerable parents
5. Recruitment through established groups, especially new parent groups, with existing social bonds and groups reaching vulnerable parents
6. Easy, “opt out” enrolment processes, with automated text reminders a week in advance reminding parents of the focus of the next session
7. Alignment of program messages with maternal and child health services, to reach those not attending, and reinforce messages for those attending the program
8. Program facilitation by maternal and child health nurses and dietitians for their specific expertise, and continuity of group facilitators to build relationships
9. Program adaptations to deliver all sessions within 12 months, to accommodate parents returning to work
10. Where feasible, providing a variety of session times (mornings, afternoons, evenings), accompanied by online program delivery and resources, to ensure a reliable source of information and to support a flexible mode of delivery (web-cam, app), especially for working parents, fathers, and geographically dispersed communities

## 6. Strengths and Limitations

A key strength of this study is the review of the whole engagement process, from program promotion, referral and enrolment, to implementation and attendance. The study is strengthened further by the inclusion of perspectives from program service providers experienced in all aspects of program implementation across three diverse geographic locations. Although the characteristics of the interviewees were comparable with those of the total sample of survey respondents, this study only interviewed English-speaking, female parents, and had a low overall response rate (10.6%). The study findings may therefore not be representative of all program participants and should be extrapolated with caution. The enablers and barriers identified in this study are similar to those in the literature, however, and therefore could be considered to have broader relevance and transferability to similar programs.

As is usual for qualitative studies, the sample size and diversity were challenged by the fact that the responding individuals were likely to be more motivated and to have been more engaged in the program, and that not all participants who expressed interest were interviewed as they were uncontactable or declined. Stratification by program attendance assisted the inclusion of multiple perspectives from non, low and high program attendees. While numbers were small, the inclusion of non-attendees also provided insights into attendance decisions for this typically under-reported cohort. 

## 7. Conclusions

The transition to parenthood is associated with a period of heightened receptiveness to information and guidance, especially in relation to infant feeding. This transition is also associated with the need for social connections and shared experiences with other parents. A group-based program delivered during the first year of parenthood providing reliable, age-appropriate messages on relevant topics such as feeding, active play, sleep and safety, such as the *Infant Program*, appears to meet this need. Maternal and child health nurses who have an established relationship with parents through routine and regular contact, provide a trusted point of referral to the program, and are in a position to positively influence attitudes to program enrolment from the first home visit after birth. Adaptations to program delivery may be necessary to extend recruitment beyond new parent groups in order to reach more vulnerable parents, where groups are more likely to have infants of mixed ages, and parents from multicultural backgrounds. 

The factors influencing parental engagement in the *Infant Program* highlight a number of personal, organisational and program barriers and enablers that are interconnected around the adjustment to parenthood and the need for knowledge, reassurances, and social connections. Identifying and understanding these factors is important so that appropriate strategies can be developed to enhance parental engagement and participation in ‘early years’ interventions.

The study findings provide important insights into the decision-making processes of new parents adjusting to parenthood and factors influencing program attendance. An exploration of the enablers and barriers experienced by program service providers advances our understanding of the implementation context and ability to support parental engagement.

## Figures and Tables

**Table 1:** Description of program implementation model elements by study site.

Study Site	Rural	Regional	Urban
**Source of referral**	Maternal and child health nurse		
**Program promotion and recruitment**	First session of *Infant Program* run at last meeting of new parent group	Short presentation at last session of new parent group	At 8-week maternal and child health visit; posters, pamphlets and flyers at new parent group venues
**Enrolment process**	Automatically enrolled into all sessions with option to opt out	Expression of interest form at last session of new parent group—enrolled for first session; responsibility of parent to enrol for subsequent sessions	Written invitation to all new parent group participants with program information; responsibility of parent to enrol for sessions
**Program sessions**	Six 1.5 h sessions at 3-monthly intervals when infant is 3–18 months of age		
**Program facilitators**	Dietitians	Social workers	Maternal and child health nurse; dietitian
**Program venues**	First session at new parent group venue; all other sessions at community health service	Range of local community venues	Range of local community venues
**Program reminders**	Text message reminder sent		

**Table 2:** Descriptive data of program participants by level of education and category of program attendance.

Program Participants by Study Site	Surveys—Number (%)	Interviews—Number (%)	School/High School Certificate		Trade/Apprenticeship Certificate/Diploma		University Degree	
			Sur.	Inter.	Sur.	Inter.	Sur.	Inter.
**Total Attendees**	53	32	6	3	17	11	30	18
**rural**	26 (49.1%)	13 (40.6%)	3	2	7	3	15	8
**regional**	7 (13.2%)	4 (12.5%)	0	0	3	1	4	3
**urban**	20 (37.7%)	15 (46.9)	3	1	7	7	11	7
**Non Attendees**	7 (13.2%)	2 (6.3%)	1	1	1	0	5	1
**rural**	1 (14.3%)	1 (50.0%)	1	1	0	0	1	0
**regional**	2 (28.6%)	1 (50.0%)	0	0	1	0	2	1
**urban**	2 (28.6%)	0 (0.0%)	0	0	0	0	2	0
**Low Attendees**	22 (41.5%)	15 (46.9%)	0	0	6	4	16	11
**rural**	9 (40.9%)	5 (33.3%)	0	0	2	1	7	4
**regional**	3 (13.6%)	2 (13.3%)	0	0	1	0	2	2
**urban**	10 (45.5%)	8 (53.3%)	0	0	3	3	7	5
**High Attendees**	24 (45.3%)	15 (46.9%)	5	2	10	7	9	6
**rural**	15 (62.5%)	7 (46.7%)	3	1	5	2	7	4
**regional**	1 (4.2%)	1 (6.7%)	0	0	1	1	0	0
**urban**	8 (33.3%)	7 (46.7%)	2	1	4	4	2	2

Sur. indicates Surveys response; Inter. indicates Interview response.

**Table 3:** Enablers and barriers to parental engagement in the *Infant Program* (personal, organisational and program factors).

Themes	Enablers	Barriers
**Transition to Parenthood (personal factors)**	Need for information as a first-time parent	Being overwhelmed
	Need for specific infant feeding information	Baby’s routine
	Need for social connections	Informal sources of information (family; internet; friends)
		Growing confidence as a parent
		Returning to work
**Program Processes (organisational factors)**	Referral through maternal and child health service	Limited awareness of program
	Recruitment via new parent groups	Recruitment of parents not attending new parent groups
	Opt-out (automatic) enrolment	Opt-in (self-referral) enrolment
	Text reminder notifications	Scheduling same-age infants
	Accessible venues	Limited session options (days/times)
**Program Processes (program factors)**	Formal sources of information (maternal and child health nurse; dietitian)	Group facilitator parenting expertise
	Group-based approach (shared experiences)	Aspects of program content
	Anticipatory guidance (timely information)	Frequency and spacing of sessions

## Supplementary Materials

The following is available online at http://www.mdpi.com/2072-6643/10/4/509/s1. Table S1: Interviewee quotes regarding enablers and barriers to parental engagement in the *Infant Program.*
