# Supplementary material for: Factors Influencing Parental Engagement in an Early Childhood Obesity Prevention Program Implemented at Scale: The Infant Program
Source: Nutrients. 2018 Apr 19;10(4):509. doi: 10.3390/nu10040509 (PMC5946294; doi:10.3390/nu10040509)
Supplement: Supplementary file 1 [file nutrients-10-00509-s001.zip › nutrients-292219-SI.pdf]

Supplementary Material Table S1: Interviewee quotes regarding enablers and barriers to parental engagement in the *Infant Program*

**THEME: TRANSITION TO PARENTHOOD**		**Program service providers**	**Non-attendees**	**Low attendees**	**High attendees**
**Enabler**	Need for information as a first time parent	----	*"Because I had no idea what I was doing. This was my first bub. It's very cliché, but babies don't come with instruction manuals…. I really wanted to get any support that I was being offered" (regional 2)*	*"I think just to gain more knowledge ...being my first child, I wasn't too sure" (urban 2)*<br>*"I think being first time mums, as well, I think everybody was pretty keen to learn and take in as much as they could about everything. I wanted to be able to, I guess, double check what I thought I knew or my instincts" (rural 19)*	*"I suppose being a first time mother just knowing different developments, how they happen, food, physical, mental, all those sort of things. A bit of reassurance" (rural 10)*
**Enabler**	Need for specific infant feeding information	----	----	*"I was feeling quite overwhelmed about introducing solids. That was probably the main reason" (urban 10)*<br>*"I was definitely interested to know about the different foods, like when to introduce the solids and what food at which stage" (rural 18)*	*"Knowing what to feed is one of the trickiest things and I was just thankful that the person running it was a dietitian because she had lots of extra information" (rural 8)*
**Enabler**	Need for social connections and shared experiences	*"I think that retention rates have a lot to do with the strength of the social bond in the group, often more than anything else" (rural 8)*	---- ----	*"There were other mothers from different new parent groups that we got to meet as well, local mothers, so that was good" (regional 3)*	*"To socialise with some of the mother's group people I know as well as other people who went …. to get out and meet other people… that sort of thing" (rural 24)*

THEME: TRANSITION TO PARENTHOOD		Program service providers	Non-attendees	Low attendees	High attendees
		"I think the people who are wanting to continue, sometimes it's a friendship thing. Yeah, in general I think when they have no issues they tend not to come but they will if they having to look forward to meeting somebody or catching up" (urban 4)<br>*"There was really good support between the Mums which was great. It's an important place to make some connections, especially if you're new to the area or something" (regional 6)*			*"I went to most of them because it was a good way to catch up and for kids to have a bit of a play. I think that was more important for some of them [participants] than the actual program itself" (rural 10)*<br>*"It was just nice to have somebody there who was going through the same thing really….. you didn't feel judged because you were all going through that, yeah, having that level of friendship really helped" (rural 14)*
**Barrier**	Being over-whelmed	----	*"I was finding it really daunting. It was very stressful…" (rural 3)*<br>*"I know it's really good to get out of the house, but I just felt—and there's so much pressure to do it. I actually felt like a bit of a failure by the end of it, but—because I wasn't getting there" (regional 2)*	---- ----	---- ----
**Barrier**	Baby's routine	----	*"It used to take me ages to get ready. When you've only got an hour fifteen wake time and it was taking me 30 minutes to breastfeed him and another ten*	*"I'd attend if it wasn't his sleep time. I wasn't going to wake him to go" (regional 3)*	*"For me, getting out at a certain time in the day, when I know that she's sleeping, got to eat, whatever… sometimes I'd be*

THEME: TRANSITION TO PARENTHOOD		Program service providers	Non-attendees	Low attendees	High attendees
			or twenty minutes to get him changed….there was hardly any time left. I used to just give up" (regional 2)		*like 'no, I'm not going'" (urban 5)*
**Barrier**	Informal sources of information (family; internet; friends)	----	*"I am open to receiving all sorts of advice. I like to take as much as possible from as many places as possible" (rural 3, year 12)*<br>*"You can Goggle all you like, there are so many forums…..but they're often full of horrible misinformation" (regional 2, post-graduate)*	*"A lot of mothers here have a Facebook group and sometimes they'll post questions on there and we'll try to answer them ourselves" (urban 7, apprenticeship)*<br>*"I usually just ask my mum… she's had four kids" (urban 2, apprenticeship)*<br>*"Most of my friends have children so I use them as a resource, so, word of mouth for me is a big thing and asking people I know" (rural 19, university)*	*"If I'm doing a Google search and it comes up with 'Raising Children's Network' I feel that that's, you know, a reliable source of information" (rural 11, university)*<br>*"My mum, who I meet every day down at the shops" (urban 17, year 12)*
**Barrier**	Growing confidence as a parent	*"I think once they have sort of got the information that they needed, particularly around the introduction of solids, and getting food going and what to feed and when, and how and all of that, once they sort of had a handle on it…. it seemed that [they felt] they'd completed the program" (regional 6)*	----	*"So, feeling a little bit more settled as a parent myself and a bit more capable and confident, you know, it just became a much lower priority" (urban 1)*<br>*"I think I just wanted to get a bit of an introductory of when I can start feeding her and I guess what kind of foods I should start introducing her to. Once I got the first bit I guess I kind of was*	----

THEME: TRANSITION TO PARENTHOOD		Program service providers	Non-attendees	Low attendees	High attendees
		"I think they're just... their thirst for knowledge in the beginning is so high because they haven't introduced food before and it's one of those scary things. Once they've attended a couple of sessions, they build their confidence in their own ability" (urban 2)		*happy just to go off and try my own thing" (urban 2)*	
**Barrier**	Return to work	*"I think that's probably one of the biggest factors, certainly most of my parents are returning to work somewhere between the time their baby is 10 and 12 months so I think that does heavily impact on the attendance at the 12 month plus sessions" (urban 2)*<br>*"And I think some of the other reasons are more like mums are going back to work in 12 months. I think we've really noticed, after the 12 months point, the 15 and 18 month sessions are pretty hard, yeah, to get people to come to" (rural 8)*	----	*"It's work. Work's the big one. If people are back at work, they just can't do it. Some mums went back to work quite early. I went back part-time at 12 months after my son was born" (regional 6)*<br>*"The first couple I was on maternity leave so they were a bit easier to get to, and then the next I was back at work at that stage so the sessions didn't always work out time-wise….everyone sort of turned up when they could…." (rural 19)*	*"I think it also got harder as well because a lot of the mothers went back to work. So, finding the time to attend, you have to take an actual day off or whatever, whereas I could fit in with my work so it didn't really matter too much" (rural 21)*

THEME: PROGRAM PROCESSES		Program service providers	Non-attendees	Low attendees	High attendees
**Enabler**	Referral through MCHN (high uptake of KAS visits)	*"I personally normally introduce the program after my eight weeks centre visit with my client, before the four month visit. Yeah, so it's the normal key age and stage visit and I chat to the parents at the visit and hand them out a flyer" (urban 4)*	----	*"I think you trust it more when it comes from a nurse, maybe. If another mum just told me about it, I probably would have half thought about it. But because the nurse knew my situation and recommended it, then that was what really made me want to go to it" (urban 11)*	*"I think it was helpful because having them [MCHN] specifically say, you know, we back this and we think it's a great program, you'll get stuff out of it. It's like equivalent to your GP recommending it because it's coming from someone that you trust" (rural 11)*<br>*"I think promoting through the nurses is a good thing, because all of the mums have to visit the nurses, routinely" (urban 16)*
**Enabler**	Recruitment via NPGs	*"I think it's probably the best way, I would say between 80 and 90% of parents going to those groups would actually express interest… and that cut out a lot of the admin of me trying to follow-up with people, because they were already coming together" (regional 5)*	----	*"It probably helped hearing about it in the [new parent] group, because I think I went along to at least the first session with some other mums from the same group" (urban 13)*<br>*"I think going to the new parent groups is perfect. You've got your captive audience, and*	*"I was in two minds as to whether I should attend or not… just chatting with the mums in my mum's group to see what they feel, whether they were planning to attend, helped" (urban 16)*<br>*"I'm not sure why I signed up now; it sounded good at the time*

THEME: PROGRAM PROCESSES		Program service providers	Non-attendees	Low attendees	High attendees
		"I think it's successful… because they're already in that group ….everyone comes along because that's what everyone else in the group is doing" (rural 9)<br>*"I'm finding when they book in, if they're in a new parent group and they've made a connection, some of them are booking in together. I think the new parent groups work well because they come together and they've got the support of each other" (urban 1)*		*they'll tell the other parents that weren't there that week, if someone wasn't there" (regional 6)*	*and others were going too" (regional 4)*<br>*"I think that worked well…. most of the mothers from my mothers' group, we sort of did it as a group…" (rural 21)*
**Enabler**	Opt-out enrolment	*"The last session of the new parent group is the first session of the program. So they have that first session and then they choose to continue with the subsequent sessions, or they choose to opt out. For us, it means that we're not wasting a*	----	----	*"The mums were put together automatically through the mother's group, yeah, it all happened automatically and I was very thankful. So it wasn't too much effort" (rural 8)*<br>*"That's why I continued. Because it was just booked in*

THEME: PROGRAM PROCESSES		Program service providers	Non-attendees	Low attendees	High attendees
		lot of time recruiting participants" (rural 8)<br>*"When I do the attendance sheet, I often get them [participants] to indicate if they're interested in attending the next session. And if they tick 'yes', then I automatically book them into that next session. I just figure it's something that might make their life a little bit easier" (urban 2)*			*and done, and I just put it in my calendar and attended the next one" (urban 4)*
**Enabler**	Text reminder notifications	*"We've had a few experiments using and not using text messages. At different times, when we've had different administrative kind of capacity, I suppose, when we don't send text messages a few days before, we don't get much of a turnout" (rural 8)*	----	*"We did get a reminder phone call as well, which was good" (regional 6)*<br>*"For me that was really good to have that text message … It was about a week's notice" (rural 18)*	*"Being contacted when it's time to attend the next one … that would ensure that I get there on the right time" (urban 9)*

THEME: PROGRAM PROCESSES		Program service providers	Non-attendees	Low attendees	High attendees
**Enabler**	Accessible venues	*"We tried to run sessions in you know, areas that were local to the families that we were recruiting" (regional 6)* *"We're on the main street in the centre of town, so there's public transport and plenty of parking. It's not a problem there" (rural 7)* *"I get participants attending from all sorts of other centres throughout the municipality so I don't think, for most families, I don't think location is a problem" (urban 2)*	*"It was in the middle of town, so it would be reasonably easy to access with most people. It's right on a bus line, so that's handy" (rural 3)*	*"It was convenient for me….there was plenty of space for everyone, so it was quite comfortable…. it was easy enough to be able to bring the pram along or you know, to have your baby there with you so you can probably try to settle them or feed them if you needed to" (urban 1)* *"If it hadn't been in town or something like that then I would have been probably less likely to attend, but the fact that it was so close to home then yes, it was good. The space was good" (regional 3)* *"Yeah they [venues] were quite easy to get to. So for me I often walked. The furthest mum away would be a 15 minute drive" (rural 1)*	*"The sessions were all very accessible for me - close, and it has plenty of room" (regional 4)* *"It was fine. There's heaps of parking out the back so that wasn't an issue. I was able to find something close to home" (urban 9)* *"It wasn't difficult to get to and very nice. There was no issue … easy to access" (rural 8)*

THEME: PROGRAM PROCESSES		Program service providers	Non-attendees	Low attendees	High attendees
**Enabler**	Trusted source of information (MCHN & dietitian)	*"The key messages are really easy for us to just roll out and easy for the parents to absorb and understand as well. I really like the content. When we did the training they said that it's probably not going to teach maternal and child health nurses anything that we don't already know, and I don't think they really did, but they certainly gave us a better way of getting that message across" (MCHN)*<br>*"Often we had quite tricky questions, I guess, that someone that wasn't a dietitian might not have known. It's potentially that we get the tricky questions because they know that we're dietitians and that we can give them that information" (dietitian)*	*"If you can have it available in other ways …. for people who are finding it hard to get out of the house. If it's online … I could have it easily accessible and if you have it up as a webpage I can bookmark it and go back to it and it can be updated and that sort of thing" (regional 2, post-graduate)*	*"I thought it [the program] felt like a reliable source versus, for example, Google, if you read things on the internet. So, I thought I might as well go - they might have some information that would be helpful" (urban 1, post-graduate)*<br>*"Sometimes it can be better from [health] professionals, than from mums who think they know everything and then you start to question that advice" (rural 1, university)*<br>*"There's so much information out there I just find it too confusing to go elsewhere, I don't know who to listen to, so I just stick with my health nurse or a health professional" (urban 10, university)*	*"I would still prefer to hear it from a nurse who has actually experienced or who has knowledge about it; rather than referring to the website" (urban 16, post-graduate)*

THEME: PROGRAM PROCESSES		Program service providers	Non-attendees	Low attendees	High attendees
**Enabler**	Anticipatory guidance (timely information)	*"They [participants] said it was an excellent way to continue basically learning what it is that their babies would be going through, and what they would be going through" (regional 5)*	----	*"I liked the idea of having the program at the relevant time. When we first went to the mother's group, they give you all the information, but you're thinking your child is only two or three months old; it's not really relevant to me just yet. That motivated me to go….to get that little bit of information when it's relevant, when it's happening" (urban 13)* *"She sort of gave us a rundown about what the next session was as well, so that way if you thought of anything in between, you had those questions ready for the session" (rural 2)*	*"It was going to be age appropriate....I remember thinking 'oh I'll be getting information that will be helpful for where we're at, at the moment'. If someone had have just thrown a book at us when the kids were born – it wouldn't have been helpful" (rural 11)* *"There're no other classes out there that have that general age-related learning for a baby's development….it [the program] gives you a good boost of the information in advance of when I'd need it, so I had time to be familiar with it, if that makes sense" (urban 9)*

THEME: PROGRAM PROCESSES		Program service providers	Non-attendees	Low attendees	High attendees
**Enabler**	Group-based approach (group discussion)	*"The real advantage of these groups is that you do build that trust, and families do feel supported" (regional 5)*<br>*"It was often a good opportunity for exchange from parent to parent, not just about us doing all the talking" (regional 6)*<br>*"I think you've got to have a bit of a flexible approach, so you've got what the session flow is, but for example if I came into a session and all the mums were happy to sit on the floor with their babies, then I might do the active play component first……" (rural 9)*<br>*"It's really about addressing what the actual parent is there for… it's really about having the flexibility in how you facilitate the session" (urban 3)*	----	*"I'm a bit of a shy person, so I liked that I could just sit there quietly but still feel part of it. There was no pressure. People asked questions; I didn't have to, because other people asked valid questions. Then there were conversations going on. It was nice to hear that everyone is on the same page as you" (urban 11)*<br>*"It was pretty relaxed, which I guess is a good thing because it gives you the opportunity to chat away about different things" (regional 3)*<br>*"It was more a general discussion, quite informal and I guess that helped because most of us knew each other….it felt like a safe place to have an honest chat about things" (rural 19)*	*"It was kind of intimate, because everyone kind of got the chance to tell their story, and chat, and ask questions, it was kind of more informal; which I found was really handy" (urban 5)*<br>*"The main thing I got out of it was listening to other people, their experiences … some real reassurance I suppose and knowing that you were in the same situation as other people, having the same week as other new mums" (rural 11)*

THEME: PROGRAM PROCESSES		Program service providers	Non-attendees	Low attendees	High attendees
**Barrier**	Awareness of program	*"Some of those mothers…they would really like to have known about the fact that this program is listed whilst they were doing their prenatal classes. It was something that perhaps, they sort of thought that down the track 'who is going to help me?'" (regional 5)*<br>*"if doctors know, or if other services know and there's that easy way to refer people, then I guess it would make it a bit easier" (rural 9)*	*"Perhaps just a little bit more information about what the sessions actually are, because maybe if I knew exactly what the sessions were it might have prompted me to try a bit harder and get out the door" (regional 2)*	*"If the pamphlet did say something about food on it, not just 'Infant Program' it might get some people's attention differently that way. Just calling it 'Infant Program' – if you read the title of the pamphlet, you don't automatically know it's going to help you with food stuff" (urban 11)*	*"It [the program] needs some more visibility. Face Book and social media is what we use mostly" (rural 23)*
**Barrier**	Recruitment of parents not attending NPGs	*"We can see that definitely does work…they come because their peers are coming, but I guess we've got the other side of it, where you're missing those that aren't already in that [new parent] group. I guess having that open option where you can add people rather than saying,*	----	*"I'd be disappointed if I missed out on info because I didn't go to mother's group that day, so think you could mail out [the program information] to everyone, that would be good" (urban 10)*	*"My sister for example, she went to her first new parent group and then hated it. She just didn't gel with the women and she never went back. So I wonder how many people miss the opportunity [of hearing about the program]" (rural 11)*

THEME: PROGRAM PROCESSES		Program service providers	Non-attendees	Low attendees	High attendees
		'Oh no, you weren't in that mums' group, you can't come'. But I guess then, for the people that are being added, they might feel excluded just because they don't know everyone" (rural 9) *"The more vulnerable parents are the ones who don't come along to the new parent's groups… it's really hard to sometimes engage them in the service to start. So getting them to voluntarily come out to a session can be quite a task in itself" (urban 3)*			
**Barrier**	Scheduling same age infants	*"Going into Supported Playgroups, programs had to be tweaked to fix what it was that they were after, because obviously you're not going to have all the children of the same age" (regional 6)*	----	*"Some of the mums from my group definitely said 'I wish I'd known about that earlier' because they'd already started [feeding solids]. For me it was alright because I didn't need it much earlier but they would have benefitted I think" (rural 1)*	*"At the 6-month session our [infants] were all like 9 or 10 months…The info provided I think was good. We were already just a little bit past that stage though" (rural 24)*

THEME: PROGRAM PROCESSES		Program service providers	Non-attendees	Low attendees	High attendees
		"I think getting a critical mass of babies at the right age is actually quite difficult…. and that is the case for a number of [rural] areas. I think you need a really good system for that, otherwise it becomes all-consuming" (rural 8)			
**Barrier**	Opt-in enrolment /self-referral	*"So it really puts the responsibility back on the parent, because what I've found is that if you book them in sometimes they don't turn up, but if you give them the responsibility and give them the choice you're more likely to see them actually turn up for the sessions. It's not just me saying right, you need to do this and being told what to do"* (urban 3) *"Organising the groups… admin-wise, it's just huge. Every one of those families that*	----	*"It's quite easy to forget. You know, you sign up when your baby's quite young and by six months and 12 months or whatever…you know it's so far past it's hard to remember"* (urban 1)	----

THEME: PROGRAM PROCESSES		Program service providers	Non-attendees	Low attendees	High attendees
		had been referred or comes to the new parent group, we had to follow-up with a phone call, and then email the information out, and then you had to do follow-up phone calls, and if they didn't answer, and sometimes they didn't have a phone, then I'd email" (regional 5)			
**Barrier**	Session options (days and times)	*"I'm still sort of thinking how we could do that… engage a bit better with fathers…as far as what their working arrangements are" (regional 5)*<br>*"I think the time of the sessions, because ours are all morning sessions. As those babies get into some of their routines as they get bigger, sometimes those times are maybe not quite flexible enough" (urban 2)*<br>*"The scheduling thing can be quite difficult. I think we found*	----	*"If you could do them after hours but still that's difficult… weekends are generally family time….evenings are hard because you're exhausted" (rural 19)*<br>*"I would have gone to the sessions if they were on a non-work day" (regional 6)*<br><br>*"It's difficult because your baby obviously does sometimes change their sleep patterns and that sort of thing…. It would*	*"They're run at a certain time, I think they were mainly mornings, and sometimes it just conflicts with my baby's schedule. And I thought maybe it would be nice to have two sessions, one in the afternoon and one in the morning, not necessarily on the same day, because that might be really difficult, but maybe in the same week. Just to give parents an option…even after-hours so dads might be able to come" (urban 5)*

THEME: PROGRAM PROCESSES		Program service providers	Non-attendees	Low attendees	High attendees
		it easiest just having a certain date so that then we could block out the diary. And if you've got staff on leave, and you've got two sessions that are competing… it makes it a bit tricky" (rural 9)		*have been nice to have a bit more options for time" (urban 1)*	
**Barrier**	Group facilitator expertise	----	----	*"For me, hearing it from facilitators who weren't mothers was a letdown" (rural 1, university)*<br>*"Parts of it were great and I got a lot out of it but there were parts where I just thought, 'I feel a bit judged now'. It was like 'this is the recommendation'" (urban 12, university)*<br>*"I already had an individual [MCHN] appointment scheduled within that week, so I didn't feel like I needed to attend the group session. When you've got the individual appointment,*	*"A few of them weren't mothers themselves, so they couldn't relate to certain things. It was a little bit like straight out of the textbook. There weren't a lot of realistic examples" (rural 10)*<br>*"I know all the facilitators are not mums, but… I think that was something that we all felt, that the facilitator didn't have the [personal] experience" (urban 16)*

THEME: PROGRAM PROCESSES		Program service providers	Non-attendees	Low attendees	High attendees
				you get to ask whatever questions you have at the time" (urban 13, university)	
**Barrier**	Aspects of program content	*"They [participants] were often quite surprised to hear the recommendations were no screen time up to two years, and there were lots of questions about 'well what do we do as parents then for our own sanity?'" (regional 6)*	----	*"It needs to be different to what they [MCHN] are covering .., because you don't want to just recreate the wheel" (regional 6, post-graduate)*<br>*"I kind of left thinking well, I didn't really get much out of that one" (urban 10, university)*<br>*"I guess I was a bit disappointed, it didn't really tell me anything that I didn't already know. I expected to get more hands on experience out of it" (regional 3, university)*<br>*"That particular [third] session was very basic… everything they told me I knew" (rural 18, university)*	*"There was no real, sort of 'This is what you should be aiming for'. It was just sort of a general discussion, which we did a lot as a mothers' group anyway. So, there was no [specific] feedback on what we should be aiming for" (rural 21)*<br>*"The first one, the three month session, was really informative, but the second session, at six months, not so much; it didn't give us much information as to what foods we could introduce to the babies at that age, and all that" (urban 16, post-graduate)*
